# An Unusual Case of Proteus mirabilis-Induced Severe Contiguous Bacterial Osteomyelitis in an Elderly Nursing Home Resident: A Case Report

**DOI:** 10.7759/cureus.57710

**Published:** 2024-04-06

**Authors:** John N McNally, Adam Bruckner

**Affiliations:** 1 Medicine, Edward Via College of Osteopathic Medicine, Spartanburg, USA; 2 Family Medicine, Aiken Regional Medical Center, Aiken, USA

**Keywords:** elderly, peripheral arterial disease, osteomyelitis, decubitus ulcers, proteus mirabilis

## Abstract

Contiguous bacterial osteomyelitis results from the spread of a variety of pyogenic bacteria from nearby skin, soft tissue, or joint infections into the underlying bone. This report describes a case of severe contiguous bacterial osteomyelitis in an 82-year-old female nursing home resident with newly diagnosed and comorbid peripheral arterial disease, along with a history of decubitus ulcers as a result of presumed neglect at her residence. The patient initially presented with multiple ulcerative lesions overlying the left foot and ankle with associated severe pain and chronic vascular insufficiency. The patient was empirically started on broad-spectrum antibiotics, with a subsequent wound culture demonstrating heavy growth of *Proteus mirabilis*. Multiple imaging modalities irrefutably demonstrated destructive bony changes characteristic of osteomyelitis. Left below-the-knee amputation was thereafter agreed upon as the most beneficial treatment method, with concomitant prolonged antibiotic therapy. This case emphasizes the importance of providing adequate medical and preventative care for elderly nursing home residents in an effort to reduce the incidence of contiguous bacterial osteomyelitis, a topic rarely discussed in current literature.

## Introduction

Contiguous bacterial osteomyelitis (CBO) can be described as a subtype of bacterial osteomyelitis in which the migration of bacteria from a nearby joint, skin, or soft tissue infection effectively seeds the underlying bony structures, resulting in an acute or chronic inflammatory process in the bone precipitated by a variety of pyogenic bacteria. The blanket term "osteomyelitis" refers generally to this acute or chronic inflammatory process. CBO may result from direct bodily trauma resulting in infected overlying skin or soft tissue, though it may also arise from the spread of bacteria from an adjacent infected decubitus ulcer most frequently associated with the lower extremities of debilitated or bed-bound patients and potential underlying diabetic neuropathy and vascular insufficiency [1].

Patients diagnosed with CBO demonstrate variability in presenting signs and symptoms based on the time since the initial infection; however, most patients typically exhibit non-specific signs and symptoms such as erythema, edema, and pain in the region of infection. Patients may also demonstrate constitutional symptoms in the more acute phase of infection, while lacking such symptoms in a more chronic form of infection [2,3]. The chronic form of osteomyelitis may also demonstrate the presence of draining sinus tracts and local signs of vascular insufficiency [4].

In general, bacterial osteomyelitis may be due to a variety of bacteria dependent on age and the nature of the infection [5]. *Staphylococcus aureus* is considered the most common causative organism in all forms of bacterial osteomyelitis, with coagulase-negative *Staphylococcus* species, gram-negative enteric bacteria, *Streptococcus* species, and anaerobes also contributing to the development of various forms of bacterial osteomyelitis in differing degrees. Notably, the gram-negative enteric bacteria *Proteus mirabilis* is considered a rarer causative organism as it relates to bacterial osteomyelitis. Osteomyelitis, as a blanket diagnosis, is said to demonstrate an incidence rate of 21.8 cases per 100,000 person-years in the United States, with a higher incidence noted among men of all ages relative to women of comparable age [6].

In this report, we describe the case of severe CBO of the left foot and ankle in an 82-year-old female nursing home resident and demonstrate the grave importance of providing proper care and medical management for our disabled elderly population, a topic rarely discussed in the context of CBO.

## Case presentation

An 82-year-old female nursing home resident with dementia presented to the emergency department via ambulance with multiple lesions noted over the left foot and ankle with associated severe pain. The patient was accompanied in the emergency department by family who provided the patient’s entire history due to her then-disoriented mental status and additional history of dementia. The patient had been previously admitted to a nursing home approximately seven years ago due to worsening dementia, with the family eventually suspecting elder neglect on the part of the nursing facility due to the development of various decubitus ulcers and the provision of an improper diet over her years as a resident. The accumulation of new and far more severe skin lesions noticed upon the family’s most recent visit prompted their request for emergency services. It was also stated that the patient had been effectively bed-bound over her tenure at her nursing home, most certainly contributing to the development of the aforementioned decubitus ulcers.

Through discussion with the family, relevant past medical history was determined to include bipolar I disorder, for which the patient was said to be taking risperidone, and type 2 diabetes mellitus (T2DM), for which the patient was prescribed insulin glargine with an incorrectly administered non-diabetic diet. The patient also had a previous history of smoking over a prolonged yet undetermined amount of time. Upon admission, the patient was said to have had multiple episodes of subjective fever over the previous several days, with the absence of concerning cardiopulmonary, gastrointestinal, or genitourinary symptoms. The family also mentioned notable bilateral lower extremity weakness and atrophy along with multiple lesions in the region of the left foot and ankle.

**Figure 1:**
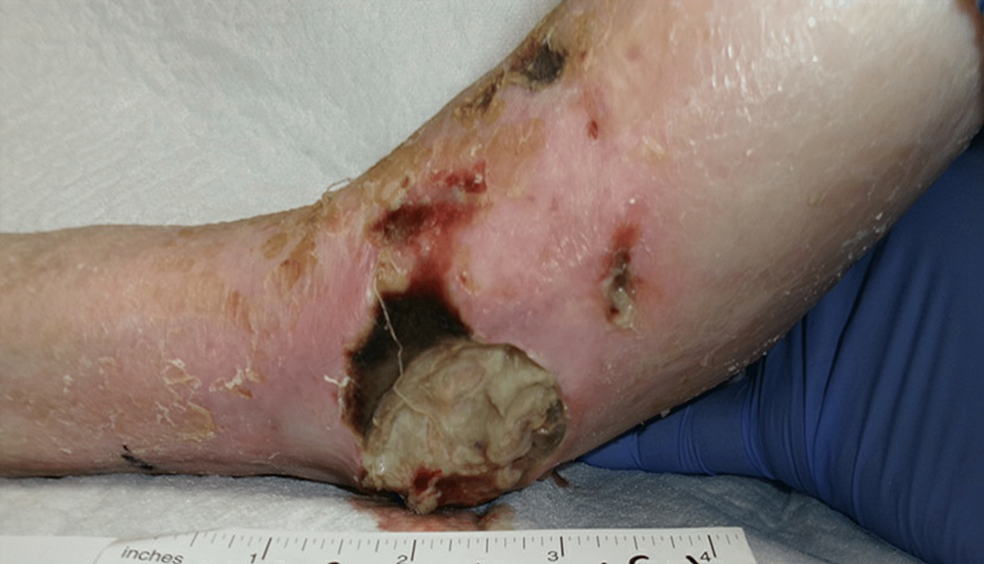
A 5-cm-wide ulcer on the anterior left ankle/foot

**Figure 2:**
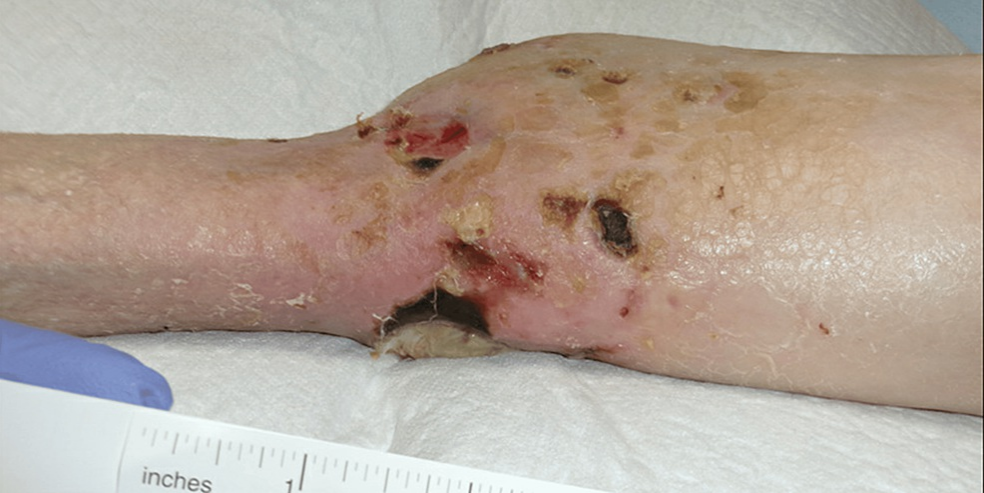
A small lesion on lateral malleolus of the left ankle/foot

**Figure 3:**
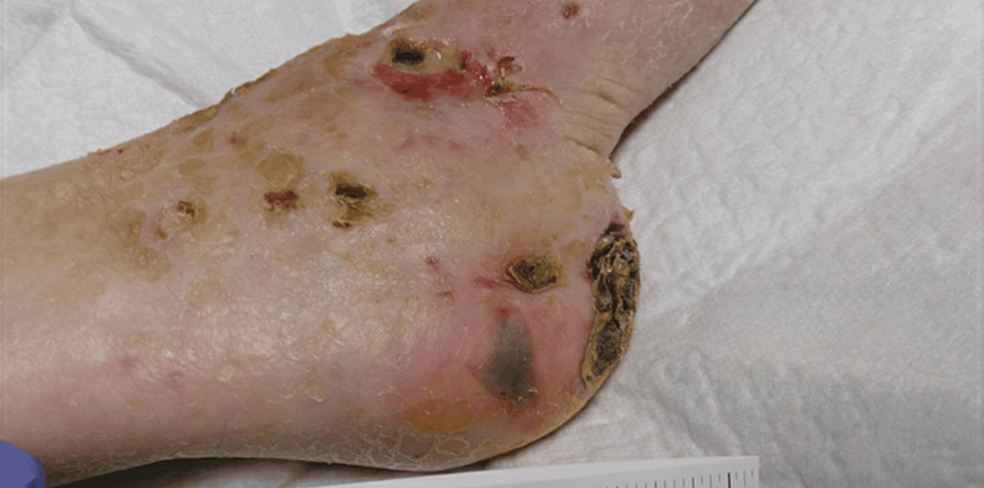
A decubitus ulcer on the posterior left heel

On physical examination of the patient, three lesions in the left foot and ankle region were noted. The first lesion was described as a 5-cm-wide ulcer with purulent and malodorous exudate present on the anterior left ankle/foot with surrounding erythema and eschar (Figure 1). The second lesion was described as a small, round lesion with the absence of drainage or eschar present on the lateral malleolus of the left ankle/foot (Figure 2). The third lesion was described as a decubitus ulcer on the posterior left heel with potential tracking to deeper tissue (Figure 3).

It was also noted upon palpation that the patient's left ankle was effectively detached from the left foot, with the foot described as hanging loosely. Additionally, an examination of the patient's bilateral lower extremities demonstrated diffuse atrophy with shiny and hairless skin; the dorsalis pedis pulse was palpable on the right but absent on the left. Neurologically, the patient demonstrated tardive dyskinesia and exhibited a disoriented mental status with a lack of orientation to person, place, or time. All additional portions of the physical exam, including admission vital signs, lacked significance. Labs on admission included a c-reactive protein (CRP) with a value of 13.2 mg/dL, white blood cell count (WBC) with a value of 12.34 x 10^3^ cells/μL, and neutrophil percentage (neutrophil %) of 76.3%, all of which indicate an inflammatory or infectious etiology; the patient’s hemoglobin A1c (HbA1c) was noted as 6.9% (Table 1).

**Table 1:** Admission laboratory values and reference ranges

Relevant Laboratory Components	Admission Laboratory Values	Reference Laboratory Ranges
C-reactive protein (CRP)	13.2 mg/dL	Normal: < 0.03 mg/dL; Minimal elevation: 0.03–1.0 mg/dL; Moderate elevation: 1.0–10.0 mg/dL; Marked elevation: >10.0 mg/dL; Severe elevation: >50.0 mg/dL
White blood cell count (WBC)	12.32 x 10^3 ^cells/µL	Normal: 4.5 x 10^3 ^to 11 x 10^3^ cells/µL
Neutrophil percentage (neutrophil %)	76.3%	Normal: 40–60%
Hemoglobin A1c (HbA1c)	6.9%	Normal: <5.7%; Prediabetes: 5.7–6.4%; Diabetes: >6.4%

Subsequent to the initial evaluation, the patient was empirically placed on 4.5 g IV piperacillin-tazobactam and 750 mg IV vancomycin to provide coverage for possible methicillin-resistant *Staphylococcus aureus *(MRSA) or *Proteus mirabilis*-related infection, with blood and wound cultures ordered to further guide proper pharmacologic treatment. An X-ray of the left ankle and MRI without contrast were both ordered as indicated for infection, with a bilateral lower extremity duplex arterial ultrasound ordered as indicated for gangrene and peripheral arterial disease (PAD).

Concerning culture results, the wound culture demonstrated heavy growth of the gram-negative enteric *Proteus mirabilis*, and the blood culture demonstrated no growth. As a result of the patient's wound culture, the empiric antibiotic regimen was left unaltered.

**Figure 4:**
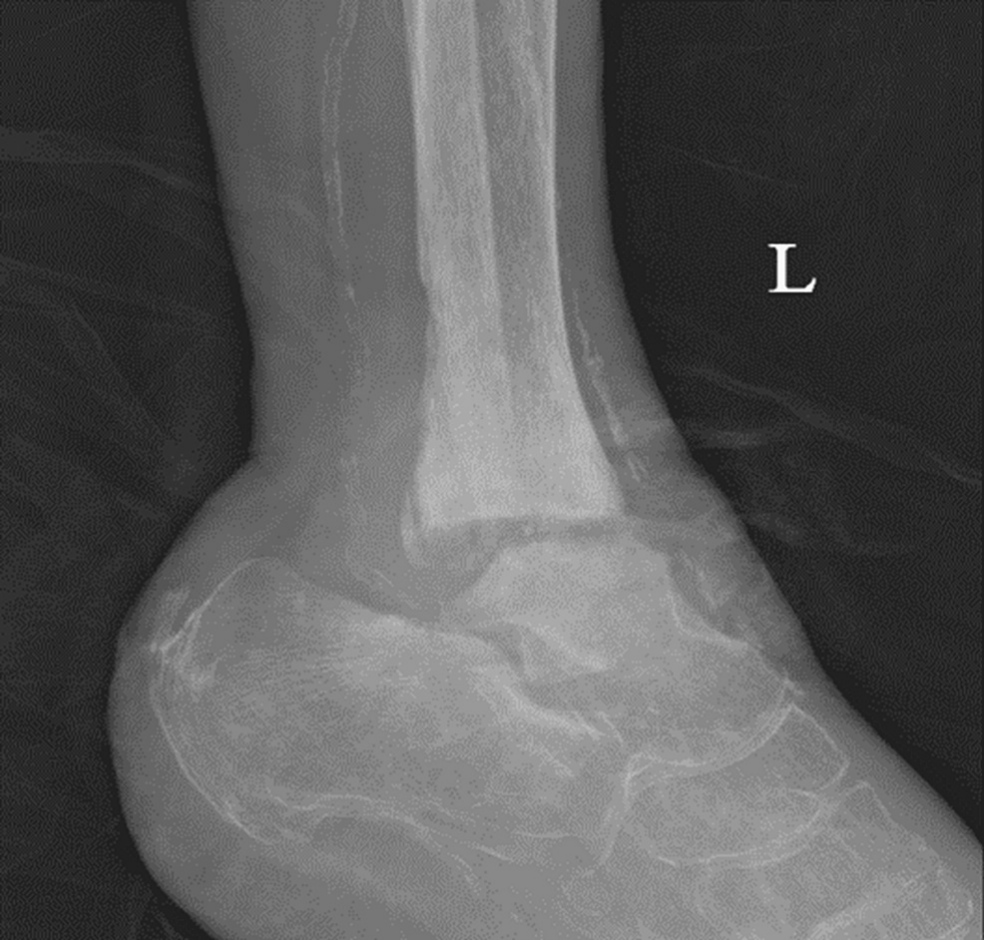
Lateral X-ray of the left ankle/foot

**Figure 5:**
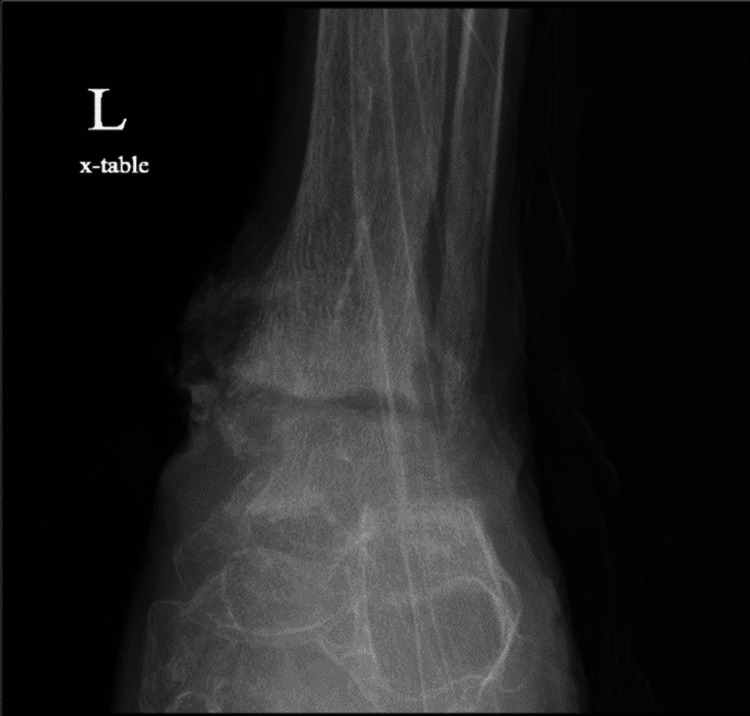
Anteroposterior X-ray of the left ankle/foot

**Figure 6:**
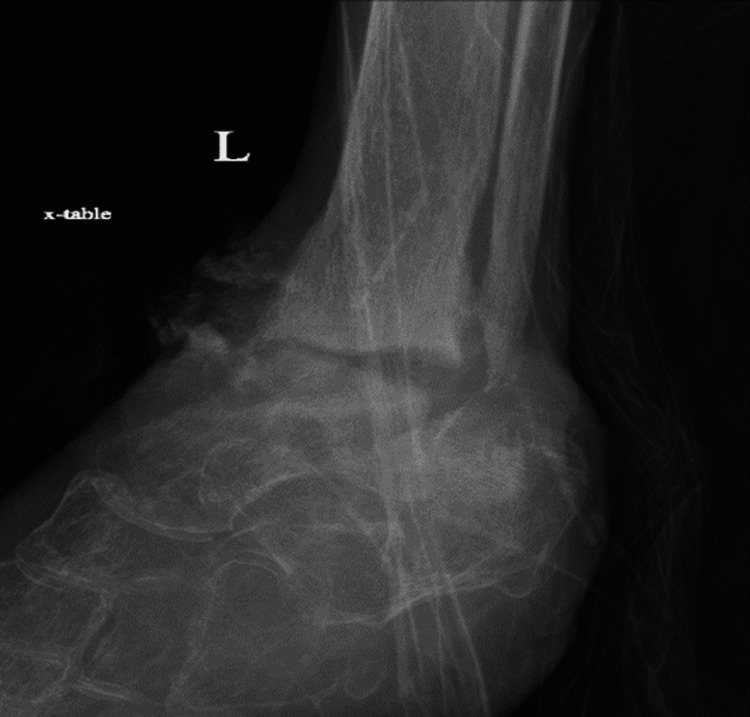
Mortise X-ray of the left ankle/foot

X-ray of the left ankle demonstrated destructive changes of the tibiotalar articular surfaces and subluxation of the talus relative to the calcaneus. The patient's X-ray of the left ankle results have been provided in the form of a lateral view (Figure 4), antero-posterior view (Figure 5), and mortise view (Figure 6).

**Figure 7:**
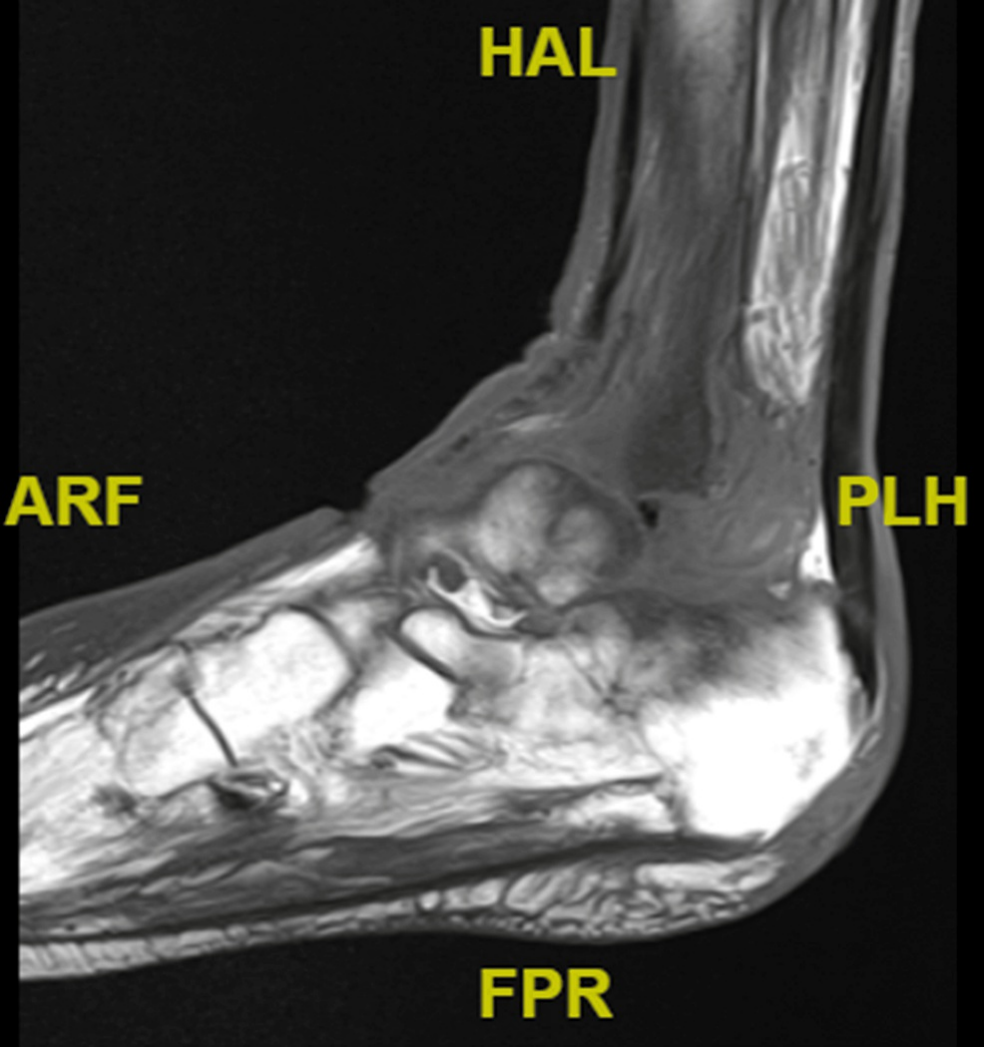
MRI without contrast of the left ankle/foot HAL: high ankle ligament, ARF: anterior radial fossa, PLH: posterior lateral heel.

Furthermore, MRI without contrast of the left ankle found a lateral malleolar wound with gas and edema within deep soft tissue, along with extensive marrow edema of the left ankle (Figure 7).

**Figure 8:**
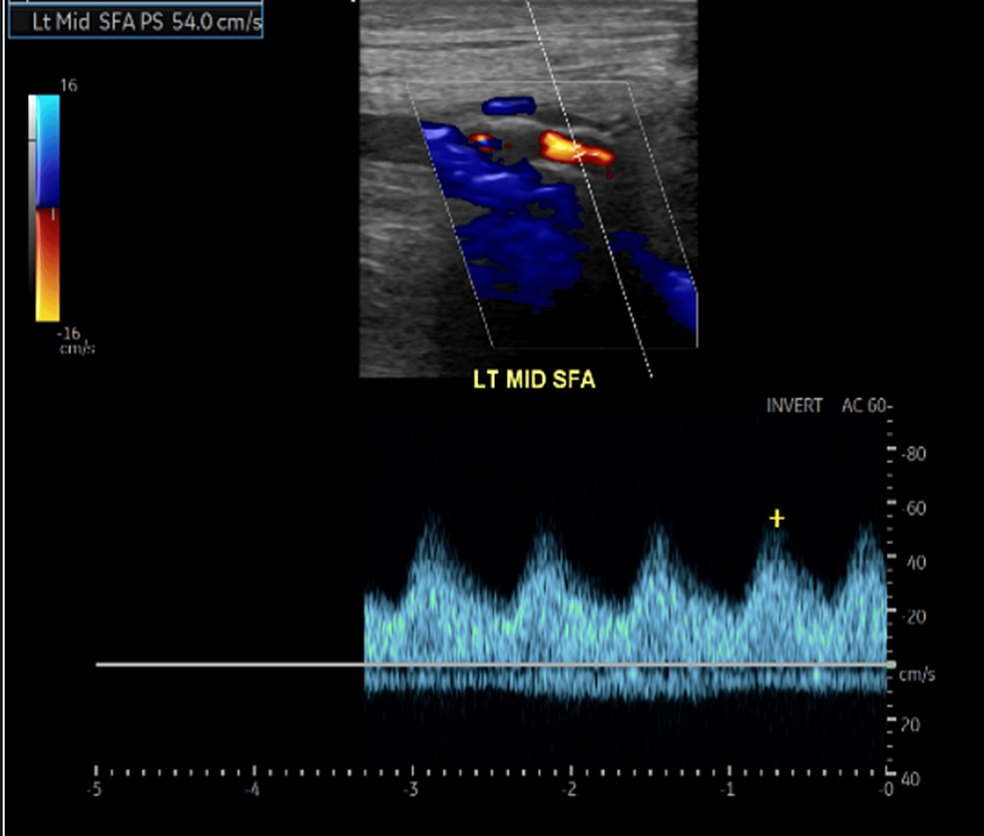
Duplex arterial ultrasound of monophasic waveforms in left mid-SFA (superficial femoral artery)

Lastly, the bilateral lower extremity duplex arterial ultrasound found hemodynamically significant stenosis of the right common femoral artery, diffuse monophasic waveforms in bilateral lower extremities, and diffuse low-velocity monophasic waveforms of the left lower extremity consistent with proximal iliac stenosis; these findings are indicative of PAD in this patient. An example of the monophasic waveforms found diffusely in this patient has been demonstrated below in the left mid-superficial femoral artery (Figure 8).

Ultimately, it was agreed upon that CBO, in this case considered diffuse, was the most appropriate primary diagnosis for this patient. PAD, as mentioned prior, was also found, but was considered a contributory diagnosis to the development of this patient’s primary diagnosis. CBO was suggested to have been a collective result of her bed-bound state, ultimately resulting in decubitus ulcer formation, and PAD, a condition impairing both immunity and healing in the affected region. The patient’s historical diagnosis of T2DM was not considered a massively contributing factor to this patient’s CBO, as her glucose levels and HbA1c were both considered to be within the well-controlled range for diabetic patients.

An orthopedic consult was performed subsequent to previous imaging and cultures with a recommendation for left below-the-knee (BKA) amputation. Unanimous agreement by the patient's family to proceed with a left BKA was made; however, the patient was immediately thereafter transferred to a healthcare facility more local to the family's residence prior to the procedure's completion. The patient's current condition is unknown due to the transfer.

## Discussion

The analysis of laboratory data, utilization of diagnostic imaging, and ascertainment of a bone biopsy are crucial components used in the evaluation of a patient with suspected CBO and are effectively essential to conclusively establish such a diagnosis. The treatment of CBO involves two primary aspects: antibiotics and surgery, which differ in degree of utilization depending on the severity of a patient’s condition [2].

Laboratory results are generally considered non-specific with regard to CBO, though the presence of leukocytosis and the elevation of inflammatory markers may be used to support the presence of infection. In the case of CBO, blood cultures acquired on admission prove to be negative, as the presence of a positive blood culture may better lend support to the alternative diagnosis of hematogenous osteomyelitis [2].

There are several forms of radiographic imaging modalities used to validate or support the diagnosis of blanket osteomyelitis; however, X-ray, MRI with or without contrast, and CT imaging are the most commonly used forms. An X-ray of the affected site is considered the most appropriate initial form of radiological evaluation and may be used to rule out potential metastatic disease or fractures; however, X-rays may be less useful in the more acute phase of osteomyelitis as it is delayed in demonstrating suggestive findings of bony infection [1,7]. MRI is considered the gold standard form of imaging to demonstrate bony changes suggestive of osteomyelitis, as it boasts the highest combined sensitivity and specificity relative to other forms of imaging, with an equally impressive negative predictive value. MRI has also been shown to demonstrate radiological evidence of osteomyelitis within three to five days of infection onset [8]. Finally, CT scans may be used when MRI is contraindicated but may also be used to guide percutaneous bone biopsies [9], as this form of biopsy has demonstrated a significantly decreased complication rate when compared to the historically performed open bone biopsies [10]. 

Furthermore, a bone biopsy is considered an essential component to establish a histopathological basis for the diagnosis of CBO, though it may be unnecessary if imaging is irrefutably demonstrative of osteomyelitis [11]. As previously mentioned, a percutaneous biopsy of the bone with guidance via CT is the currently preferred method to obtain a bone sample. When compared to open bone biopsies, the percutaneous modality is preferred due to heightened patient safety and decreased morbidity [10].

Regarding the treatment of CBO in this case complicated by PAD, prolonged use of broad-spectrum antibiotics including coverage for anaerobic bacteria and MRSA may be initiated empirically, with bone biopsy results later guiding further pharmacologic treatment decisions. Evaluation of the degree of a patient’s vascular insufficiency to determine the salvageability of the affected limb may be performed with duplex arterial ultrasound or ankle-brachial index. In the event an affected limb exhibits extensive necrosis or gangrene, partial or complete amputation is considered an appropriate treatment option. If the affected limb demonstrates significant ischemia but lacks diffuse bone involvement or the presence of extensive gangrene or necrosis, arterial bypass procedures may be performed to improve the potential for limb salvage, though they are still followed by surgical debridement of present necrotic tissue [2].

With this being said, adequate precautions must be taken in nursing facilities to prevent the development of decubitus ulcers and to limit the worsening or development of vascular insufficiency. The use of specially designed beds with static surfaces, such as fluid-, air-, or foam-filled mattresses, or even beds with dynamic surfaces, such as the pneumatic ripple bed, may be effectively used to eliminate pressure to regions of the body involving bony protuberances at high risk of ulcer formation when compressed. Specially designed cushions may be used for wheelchair-bound patients and include gel and pneumatic components that distribute the load in a more balanced fashion over these same bodily regions. Additionally, the use of protective devices encasing or providing support for the feet and lower extremities may decrease the risk of ulcer formation in these regions [12]. The risk of developing PAD in nursing homes may be partially prevented by smoking cessation, obtaining adequate physical activity, and with proper control of pre-existing hypertension, dyslipidemia, and diabetes mellitus. In a patient already diagnosed with PAD, a multifaceted approach including the aforementioned prevention strategies in combination with anti-platelet or anticoagulation therapy, peripheral vasodilators, and a supervised exercise training program may be utilized [13].

## Conclusions

CBO is a serious medical condition that may develop due to the spread of bacteria from a nearby skin or soft tissue infection seeding underlying bone. Of importance to this case, infected decubitus ulcers in conjunction with co-existing PAD may play a role in the development of CBO in neglected and disabled elderly individuals. Though surgical and pharmacologic interventions are available for the treatment of this condition once diagnosed, preventing the development of decubitus ulcers, managing preexisting medical conditions, and preventing or limiting the progression of vascular insufficiency are important measures that must be taken to prevent the development of CBO in the first place.
